# Fatal Myocarditis in Course of *Plasmodium falciparum* Infection: Case Report and Review of Cardiac Complications in Malaria

**DOI:** 10.1155/2011/202083

**Published:** 2011-04-14

**Authors:** Paola Costenaro, Paolo Benedetti, Chiara Facchin, Carlo Mengoli, Giampietro Pellizzer

**Affiliations:** ^1^Scuola di Specializzazione in Malattie Infettive, Università degli Studi di Padova, Via Giustiniani, 35122 Padova, Italy; ^2^Unità Operativa di Malattie infettive e Tropicali, Ospedale S. Bortolo, Viale F. Rodolfi 37, 36100 Vicenza, Italy

## Abstract

We describe a fatal case of imported malaria where the sole finding revealed at the *postmortem* evaluation was an acute lymphocytic myocarditis with myocardiolysis. This case recalls the potential importance of myocardial injury in the prognosis of malaria and prompts a reevaluation of current perspectives on the pathogenesis of severe falciparum infection. In the light of this, we have reviewed the cases of cardiac complications in malaria published to date.

## 1. Introduction

Malaria still remains one of the major health problems especially in developing countries. An estimated 250 million cases led to nearly one million deaths in 2006, mostly of children under 5 years [1]. If not recognized promptly, *P. falciparum* malaria can in fact retain a high case-fatality rate, especially in nonimmune persons. Cerebral malaria remains the most common clinical presentation and cause of death [2]. In contrast, myocardial failure and cardiac arrhythmias have been rarely reported in course of severe malaria despite the well known sequestration of parasitized erythrocytes in the myocardial vessels and the potential cardiac toxicity of antimalarial drugs. However, primary myocardial involvement has recently been observed in a few cases of imported severe falciparum malaria; all of these cases exhibited a particularly severe clinical course, and two deceases were recorded [3, 4]. We describe a fatal case of malaria in which the primary cause of death revealed by the *post mortem* evaluation was acute myocarditis.

## 2. Case Presentation

**Figure 1:**
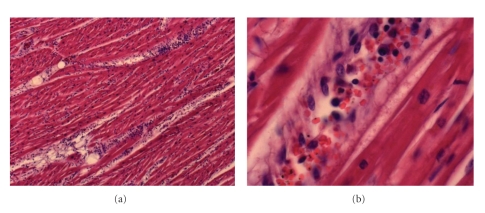
Acute myocarditis due to *P. falciparum*. (a) Extensive lymphocytic infiltrates surrounding myocardial capillaries and (b) Lymphocytes and parasitized RBCs sequestered in the lumen. Haematoxylin and eosin, ×20 (a) and ×40 (b); original magnification.

A 50-year-old Italian man was admitted to the hospital because of severe febrile illness associated with profound malaise, headache, sweating, and worsening jaundice of five-day duration. Two weeks before the onset of sickness he had returned from a business trip in Cameroon. The patient had not taken any chemoprophylaxis against malaria. He was obese (weight 98 Kg; height 176 cm), and an excessive use of alcohol was reported. He had not previously suffered from hypertension or any other known cardiovascular disease. Upon admission, he complained additionally of nausea, vomiting, and diarrhoea. He was fully conscious, normotensive with a blood pressure of 120/80 mm/Hg, but clinical examination revealed tachycardia and severe jaundice; his peripheral oxygen saturation was 95%. A neurological inspection did not show significant alterations. Full blood count revealed thrombocytopenia (17 × 10^9^/L), haemoglobin at 14.6 g/dL, and white cell count at 10 × 10^9^/L. Liver function tests documented hyperbilirubinaemia, moderate increase in serum transaminases, and moderate renal impairment; blood glucose levels were also raised. Coagulation screen did not show evidence of disseminated intravascular coagulation. Cardiac enzymes were normal (Table 1). A blood film revealed a heavy *P. falciparum* parasitaemia of 20%. A chest X-ray was normal, and the unique alteration observed in the electrocardiogram (ECG) was a low amplitude in QRS complexes. Serological testing for *Cytomegalovirus*, *Epstein-Barr virus*, seasonal influenza viruses A and B, adenoviruses, *Chlamydia psittaci, Coxiella burnetii, Mycoplasma pneumoniae*, respiratory syncytial virus, the Widal-Wright reaction and the slide macro-agglutination test for leptospirosis were negative. The patient was treated with 10 mg/kg b.w. (namely, 1000 mg) of I.V. quinidine gluconate t.i.d., plus ceftriaxone 2 g q24h I.V. and oral doxycycline 100 mg b.i.d., while waiting for the results of blood cultures. Within 12 hours of admission, a slow but progressive worsening of multiorgan failure was observed despite a marked reduction of parasitaemia. Given the severity of clinical setting, I.M. artemisin (300 mg loading dose, followed by 100 mg/daily) was initiated. At 40 hours after admission, the patient worsened suddenly. He became restless, confused, bradiarythmical, hypotensive (80/60 mmHg), and died in spite of prompt mechanical ventilation and cardiac resuscitation. The *post-mortem* evaluation revealed acute heart failure (bilateral pulmonary oedema) but no signs of myocardial ischaemia. The liver and the spleen were not enlarged. However, an histology of severe acute myocarditis with myocardiolysis was detected: diffuse lymphocytic infiltrates were surrounding the myocardial capillaries, inside which some intravascular parasitized RBCs were visible (Figure 1). No sequestered parasites could be revealed in brain vessels.

## 3. Discussion

According to the WHO criteria, severe *P. falciparum* malaria in adults is defined by one or more of the following: impaired consciousness with unarousable coma, jaundice, progressive renal impairment, metabolic acidosis, hyperlactataemia and hypoglycaemia, respiratory distress, pulmonary oedema and severe anaemia. The pathogenetic mechanism is believed to consist mainly of impaired tissue perfusion resulting in hypoxaemia and metabolic acidosis. Primary cardiac involvement is thought to be rare and myocardial function preserved even in severe disease [2, 5–7]. Haemodynamic changes have been found to be compatible with systemic and pulmonary vasodilation, and increased pulmonary vascular permeability to be the cause of pulmonary oedema [8]. Nevertheless, there are some reports of myocardial involvement [3, 4, 9–13] (Table 2), sometimes associated with a fatal outcome.

A raised cardiac index has repeatedly been observed in patients with severe malaria, and proposed to depend on the cytokine-mediated low vascular resistance triggered by parasite-derived pyrogens [8, 14]. There was also evidence, in complicated *P. falciparum* malaria cases compared to uncomplicated cases [15], of a significant increase in the level of N-terminal probrain natriuretic peptide (NT proBNP, a sensitive marker of impaired left ventricular function), heart-type fatty acid-binding protein (H-FABP, a marker of acute myocardial injury), myoglobin and creatine kinase muscle-brain (CK-MB) (both established markers of myocardial injury and necrosis) even in patients who did not display significant ECG abnormalities. In another study, the serum concentration of cardiac troponin T was found to be elevated only in in a very low (0.6%) proportion of patients [7], although ECG aspecific abnormalities—such as, delayed conduction and/or T or ST alterations—were observed in 14.3% of patients, suggesting that the electrophysiology of cardial myocites can be altered before myocytolysis occurs.

Autopsy data supports the view that the mechanical blockage of capillaries exerted by malarial parasites and parasitized red blood cells (PRBCs) can lead to ischaemic cardiomyopathy [4, 9, 11, 16]; the severity of clinical features was thus put in relation with the high burden of PRBCs, which exhibited an increased ability to sequester in the deep microvasculature [17]. However, in two fatal cases of *P. falciparum* infection the only significant finding detected at *post-mortem* evaluation was an acute lymphocytic myocarditis [4]. More recently, myocarditis was also observed as a complication of *P. vivax* infection [12]. Therefore, a reevaluation of current perspectives on the pathophysiology of myocardial dysfunction in course of severe malaria appears to be necessary. Toxic effects due to cytokines such as the tumor necrosis factor (TNF), have been claimed to play an important role [18–20]. An overexpression of caspases and calpains—which are believed to be inducers of apoptosis—in the presence of PRBCs and TNF described in a report [21], was not confirmed subsequently [22]. Instead, plasmodial glycosylphosphatidylinositol (GPI)—either free or linked to surface antigens—was proven, in a murine model, to retain a direct effect (i.e., independent from cytokine production by monocytes) on cardiac myocytes [23]. More recently, such an effect was determined as an upregulation of apoptotic genes and of a myocardial damage marker (NT proBNP), suggesting that GPI might induce myocyte apoptosis and therefore be one cause of malaria myocarditis [11]. In summary, at the present state of knowledge myocardial damage appears to retain a multifactorial pathogenesis, being probably the result of mechanical (microcirculatory obstruction), metabolic (systemic acidosis and related tissue hypo-oxigenation), and humoral mechanisms. However, cardiac side effects related to therapy should also be considered. Quinine may evoke arrythmias, angina, and hypotension, potentially causing circulatory failure and/or cardiac arrest [14]. However, these effects are rare and generally occur when the drug is injected rapidly: noticeably, cardiovascular collapse is generally an effect of acute toxicity manifesting when infusion is initiated [2, 24]. None of these side effects could be ascertained in our case, nor could the autopsy reveal any organ abnormality potentially in relation with the cardiotoxicity of quinine, which had been administered in a currently accepted dosage (i.e., loading dose of 15–20 mg/kg followed by 10 mg/kg t.i.d.). The experience on quinine dosing in obesity is limited, and dose adjustment for renal impairment is not recommended within the first 48 hours of treatment, since its metabolism is thought to be mainly hepatic [25]. To date, there is no direct evidence for significant cardiovascular effects of artesunate [25, 26], although a case of limited myocardial necrosis occurring just after completion of antimalarial treatment with artemether/lumefantrine was recently reported in an experimentally infected healthy volunteer, raising an issue of differential diagnosis between acute coronary syndrome and myocarditis [27]. 

In our case, additional risk factors for cardiomyopathy, such as obesity and increased alcohol intake, might have contributed to the severity of the disease. However, in spite of the severity of his clinical presentation, our patient did not develop any impairment of consciousness or other neurological symptoms. Instead, he had a clinical picture of multiple-organ failure with metabolic acidosis, and in a few hours deteriorated despite supportive and specific antimalarial treatment. The myocarditis, revealed by post-mortem histology, was unexpected. The pathological finding of active lymphocytic myocarditis usually correlates with either acute myocardial infarction-like syndrome (with normal coronary arteries) or heart failure, with normal-sized or dilated left ventricle and haemodynamic compromise [28]. Interestingly, in our patient the second scenario appears to be the most likely, even though it was so far documented in cases who had exhibited much longer courses of disease. 

Our experience suggests that in course of severe *P. falciparum* malaria the frequency of primary cardiac complications may be underestimated, especially in adult patients with cardiovascular risk factors (i.e., obesity, smoking, diabetes, hypertension, advanced age), but also in case of unknown or silent underlying cardiomyopathy. 

## Figures and Tables

**Table 1:** Patient biochemical parameters detected during hospital stay.

	Normal range	Day 1	Day 2	Day 3
		(5:30 PM)	(7:00 AM)	(6:00 AM)
WBC (×10^9^/L)	3.5–11.0	9.0	17.7	23.8
RBC (×10^12^/L)	4.1–5.65	3.84	2.93	2.90
Haemoglobin (g/dL)	12.5–16.9	12.6	9.3	9.0
PLT (×10^9^/L)	110–330	17	37	59
Creatinine (mg/dL)	<1.3	2,58	3.72	5,24
GFR (mm/h)	>90	26	17	11
Glycaemia (mg/dL)	60–126	167	144	157
Sodium (mmol/L)	135–145	136	137	143
Calcium (mg/dL)	8.5–10.5	8.1	7.7	7.8
Potassium (mmol/L)	3.3–5.0	4	4.3	4
Chloride (mmol/L)	95–110	100	102	104
Lactic dehydrogenase (lUlL)	200–420	1951	—	2479
AST (lUlL)	<37	129	110	108
ALT (lUlL)	<53	115	71	76
Total bilirubin (mg/dL)	0.3–1.5	11.5	23.5	23.5
Direct bilirubin (mg/dL)	0.1–0.6	6.5	14.8	14.8
Creatine phosphokinase (lUlL)	<200	89	74	—
Troponin (ng/mL)	<0.07	0.0	—	—
Myoglobin (ng/mL)	<110	91	—	—
PT (seconds)	11.0–13.5	11	—	12
PTT (seconds)	25–38	36	—	33
INR	0.7–1.2	1.1	—	1.2
Fibrinogen (mg/dL)	200–400	384	—	577

**Table 2:** Cases of cardiac complications in severe malaria (reported as of January 2011).

Reference	No. of cases	Organism	Concomitant morbidity	Clinical setting	Outcome
Herrera [9]	1	*P. vivax*	None	ischaemic myocarditis	Death
Mohsen et al. [3]	1	*P. falciparum*	None	acute myocarditis	Cure
Wichmann et al. [4]	2	*P. falciparum*	Unknown	myocarditis (1 pt) unknown (1 pt)	Death Death
Tripathy et al. [10]	1	*P. falciparum*	N/A	myocarditis	N/A
Wennicke et al. [11]	1	*P. falciparum*	Unknown	acute heart failure	Death
Kim et al. [12]	1	*P. vivax*	None	myocarditis	Cure
Kumar et al. [13]	2	*P. falciparum*	None	acute heart failure (1 pt) ventricular fibrillation (1pt)	Cure Death
Present case	1	*P. falciparum*	Obesity, increased alcohol intake	myocarditis	Death
